# Antimicrobial analysis of honey against *Staphylococcus aureus* isolates from wound, ADMET properties of its bioactive compounds and in-silico evaluation against dihydropteroate synthase

**DOI:** 10.1186/s12906-023-03841-z

**Published:** 2023-02-06

**Authors:** Uwem Okon Edet, Elizabeth Nkagafel Mbim, Esu Ezeani, Okoroiwu Uchechi Henshaw, Oju R. Ibor, Ini Ubi Bassey, Edet Effiong Asanga, Ekpo Eyo Antai, Francisca O. Nwaokorie, Bassey Okon Edet, Glory P. Bebia, Curtis Tega, Clement I. Mboto, Ani Nkang, Ada Francesca Nneoyi-Egbe

**Affiliations:** 1 Department of Biological Science, Faculty of Natural and Applied Sciences, Arthur Jarvis University, Cross River State, Akpabuyo, Nigeria; 2 Department of Public Health, Faculty of Basic Medical Sciences, Arthur Jarvis University, Cross River State, Akpabuyo, Nigeria; 3 The Medical Research Council Unit The Gambia at The London, School of Hygiene and Tropical Medicine, London, UK; 4 Department of Medical Laboratory Science, Faculty of Basic Medical Sciences, Arthur Jarvis University, Cross River State, Akpabuyo, Nigeria; 5 Department of Zoology and Environmental Biology, Faculty of Biological Sciences, University of Calabar, Cross River State, Calabar, Nigeria; 6 Department of Microbiology, Faculty of Biological Sciences, University of Calabar, Cross River State, Calabar, Nigeria; 7 Department of Chemical Sciences (Biochemistry Unit), Faculty of Natural and Applied Sciences, Arthur Jarvis University, Cross River State, Akpabuyo, Nigeria; 8 Department of Biological (Microbiology) Oceanography, Faculty of Oceanography, University of Calabar, Nigeria. (Antai), Calabar, Nigeria; 9 Department of Medical Laboratory Science, College of Medicine, University of Lagos, Nigeria, Lagos State, Nigeria; 10 Department of Medical Microbiology/Parasitology, Faculty of Medical Laboratory Science, University of Calabar (Glory), Calabar, Nigeria; 11 Microbiology Department, Faculty of Biological Sciences, University of Calabar, Calabar, Nigeria; 12 Department of Biochemistry, Faculty of Basic Medical Sciences, University of Calabar, Calabar, Nigeria

**Keywords:** Molecular docking, ADMET, MDR, Honey, Dihydropteroate synthase, *S. aureus*

## Abstract

**Background:**

One of the main challenges of wound healing is infection with multi-drug resistant (MDR) bacteria such as *Staphylococcus aureus.* The spectrum of antibiotics used to treat them is declining; thus, there is a need for alternatives. Our study was designed to evaluate the antimicrobial properties of honey, its pharmacokinetics (ADMET) properties and in-silico analysis of its bioactive compounds against dihydropteroate synthase of *S. aureus* using trimethoprim as control.

**Methods:**

Standard protocols were employed in collection and preparation of samples, generation of canonical strings, and conduction of microbiological analyses. Bioactive compounds’ ADMET properties were evaluated using the SWISSADME and the MCULE toxicity checker tools. The MCULE one-click docking tool was used in carrying out the dockings.

**Results:**

The gas chromatography-mass spectrophotometry revealed twenty (20) bioactive compounds and was dominated by sugars (> 60%). We isolated a total of 47 *S. aureus* isolates from the wound samples. At lower concentrations, resistance to trimethoprim (95.74 to 100.00%) was higher than honey (70.21 to 96.36%). Only seven (7) isolates meet Lipinski’s rule of five and ADMET properties. The docking scores of the bioactive compounds ranged from -3.3 to -4.6 while that of trimethoprim was -6.1, indicating better binding or interaction with the dihydropteroate synthase. The bioactive compounds were not substrates to P450 cytochrome enzymes (CYP1A2, CYP2CI9 and CYP2D6) and p-glycoprotein, indicating better gastrointestinal tract (GIT) absorption.

**Conclusion:**

The favourable docking properties shown by the bioactive compounds suggest they could be lead compounds for newer antimetabolites for management of MDR *S. aureus*.

**Supplementary Information:**

The online version contains supplementary material available at 10.1186/s12906-023-03841-z.

## Background

Wound healing is a biological process that comprises of different stages namely inflammation (platelet accumulation, coagulation and leukocyte migration), tissue formation (re-epithelialization, angiogenesis, fibroplasia and wound contraction) and tissue remodeling. Various compounds such as soluble mediators, extracellular matrix, and parenchymal blood cells are also involved in the process [1, 2]. Wound healing is a natural process and for any wound to heal, all these phases must occur sequentially and within a time frame. Any interference in the process will impair the wound healing process. [3]. The factors that interfere include those that are local or intrinsic to the wound or extrinsic. These factors include age, desiccation, infection or abnormal bacterial presence, maceration, necrosis, pressure, trauma, body type, underlying chronic diseases, nutritional status, and vascular irregularities [4]. Others include, the presence of underlying illnesses such as diabetes [5, 6]. Among these factors, bacteria presence stands out as they can cause infection, a single process that can interfere with the entire healing process [5].

Infection is an extrinsic factor capable of strongly retarding the entire healing process. The presence of live bacteria and their toxins are known to bring about a strong upregulation and prolonged activity of pro-inflammatory cytokines, excessive inflammatory responses and damage to the affected tissue [5, 6]. The increased presence of the inflammatory cytokines, bacterial load and their products drive the over expression of matrix metalloprotease that degrades the extracellular matrix and, in the process, delays wound healing [5, 6]. As revealed by Bowler et al*.* [7], the sources of microbes include the surrounding environment and surrounding skin (normal skin flora). Different microbes are known to infect wounds [7–9] and are commonplace in chronic wounds [10].

A chronic wound is a common complication in diabetics and other underlying health conditions such as poor nutrition. It presents with poor clinical outcomes, delayed wound healing, high morbidity and increased burdens (economic and health-care) to patients and governments [11]. Falcone et al*.* [11], utilized shotgun metagenomics to examine the microbiome of wounds of persons with diabetes. Their results revealed strain-level variations among *Staphylococcus aureus* [11]. *S. aureus,* especially the multidrug-resistant strain is on the World Health Organization’s list of priority pathogens for which antibiotics are highly needed [12, 13]. *S. aureus* is among the top four colonizers of chronic wounds. Its outstanding abilities include the formation of biofilms and the acquisition of multidrug resistance (MDR) genes with ease [10, 14]. *S. aureus* biofilms cause deficiencies in granulation tissue collagen [14]. Chronic wounds are difficult to treat or manage. One of the reasons is the widespread sharing of resistance genes [10]. Kalan et al*.* [10] showed that debridement rather than antibiotics therapy significantly shifted species and brought about better clinical outcomes. The challenge of MDR has driven the search for newer and safe alternatives to antibiotics [15]. Such an alternative includes using honey [15] and medicinal plants [3]. Honey is a natural product made from the nectar of flowers collected by bees and stored in their honeycomb [16]. Honey has many medicinal uses locally and these include the management of cough, sugar level and wounds [17].

Honey composition varies from one location to another. Generally, it contains sugars, amino and organic acids, vitamins, enzymes, water (> 80%), phytochemicals and antioxidants, among others [16]. Phytochemicals have well-established antimicrobial properties [13]. Collectively, these properties and products improve the wound healing properties of honey [2, 14] and other medicinal properties [17]. With the spectrum of effective antibiotics narrowing every day, phytochemicals hold great promise for newer antibiotics against MDR pathogens [13, 18, 19], especially with approaches such as molecular docking [19]. Prokaryotes elaborate several enzymes and proteins that are targeted for new drug development. Dihydropteroate synthase is unique to prokaryotes and is involved in the synthesis of folic acid, a precursor for the synthesis of nucleic acid. It is the target of sulfa and trimethoprim-like drugs, a class of drugs for which resistance has been widely reported [18]. In this study, we evaluated the in-vitro activity of *S. aureus* isolates obtained from chronic wounds and also their bioactive compounds against the dihydropteroate synthase of *S. aureus*.

## Methods

### Collection of honey

The honey was collected fresh and transported within 12 h to the laboratory. The honey (4 L) used in the study was collected aseptically from a bee farmer in northern Cross River State (Obudu), Nigeria. It was identified and assigned the validation number AJU/MCB/06/21. The honey sample was then stored at 2–8 °C for later use.

### Inclusion and exclusion criteria

The inclusion criteria set in this study included willingness to participate (informed consent oral and written), the presence of any festering wound on the leg (diameter ≥ 2 cm) and age limit ≥ 18 years (the mean age of the respondents was 41.50 ± 16.66 years). The male (*n* = 3) to female (*n* = 3) ratio was 1:1. Wound was suspected to be infected if they were purulent, becoming wider and not healing [20]. Participants who were using honey in the management of their wounds were excluded from the study.

### Collection of wound swabs

Six participants were recruited into the study that met with the inclusion criteria set and also gave informed consent. All the participant were attending Arthur Jarvis University Hospital between September and December 2020. For each patient, after cleaning the wound surface with sterile water, one swab stick per patient was used to collect wound sample by rolling over the swab stick gently over the entire surface of the wound. The swabs were immediately transported to the laboratory. Collection of wound samples was done by a registered nurse and as previously reported [20].

### Microbiological analysis

#### Sterility test

The honey sample was subjected to sterility test for certainty and this was done as reported previously [21] but with little modification. The honey sample was filtered using a sterile metal mesh (3 mm in diameter). Following filtration, a 10-serial dilution was done using 1 ml aliquot from the stock sample. From various (2^nd^,3^rd^ and 4^th^) dilutions as well as the stock solution, 1 ml aliquot was pour-plated on freshly prepared chocolate (incubated anaerobically), blood and MacConkey’s (incubated aerobically) agar plates. After overnight incubation, the plates were examined for growth. The absence of growth after 24–48 h affirmed the sterility of the honey sample.

#### Purification and biochemical identification of isolates

The wound swabs were streaked onto prepared nutrient agar and Mannitol salt agar (MSA) using a zig-zag pattern and at the same ensuring that all the sides of the swab’s stick are used in the streaking process. The plates were incubated at 37 °C for 24 h. Following incubation, discrete colonies were sub-cultured unto freshly prepared nutrient and MSA plates twice to purify the isolates. Pure isolates were stocked in sterile bijoux bottles for identification and other uses. *S. aureus* species were morphologically identified via their distinctive golden yellow pigmentation of MSA plates. Furthermore, identification was done using methods previously reported [13, 22].

#### Antibacterial susceptibility of honey

The antibacterial activity of the honey was evaluated following the agar-well diffusion method [13, 22, 23]. Briefly, the *S. aureus* isolates were sub-cultured using nutrient agar and incubated for 18 h at 37 °C. The purified isolates were suspended in peptone water and adjusted to MacFarland standard (10^7^cells/ml). The standard inoculum was then used to flood freshly prepared Mueller–Hinton agar plates, drained and allowed to stand for 1 h. Exactly, six-millimeter diameter wells (three per plates) were made in each of the agar plates using a sterile borer. Various concentrations (100% (neat) 50%, 25%, 12.5%, 6.25%, 3.13%, 1.57%, 0.78% and 0.39%) of the honey were prepared using doubling dilution technique. Similarly, using sterile distilled water as diluent, 100 mg of trimethoprim was also diluted down to 0.39 mg/ml. One hundred microliter of each of the trimethoprim and honey dilutions were added to the wells while 100 µL of phosphate-buffered saline were used as positive control. The solutions were incubated at 35 °C for 24 h. The zones of inhibition were measured in millimetres and interpreted as reported previously [22].

### GC–MS analysis of the honey

Ten (10) ml of the honey sample was added to 20 ml of methanol, the mixture shaken for 15 min and allowed to stand for another 15 min. Thereafter, the mixture was transferred to a rotatory evaporator and concentrated to 20 ml. Screening for bioactive compounds was done using an Agilent 5890 N gas chromatography equipped with an autosampler connected to an Agilent mass spectrophotometric detector. All operating condition were same as previously reported. The identification time was based on retention time [23]. The interpretation of GC–MS was conducted using the database of National Institute Standard and technique (NIST) [23].

### Retrieval of proteins and ligands

**Fig. 1:**
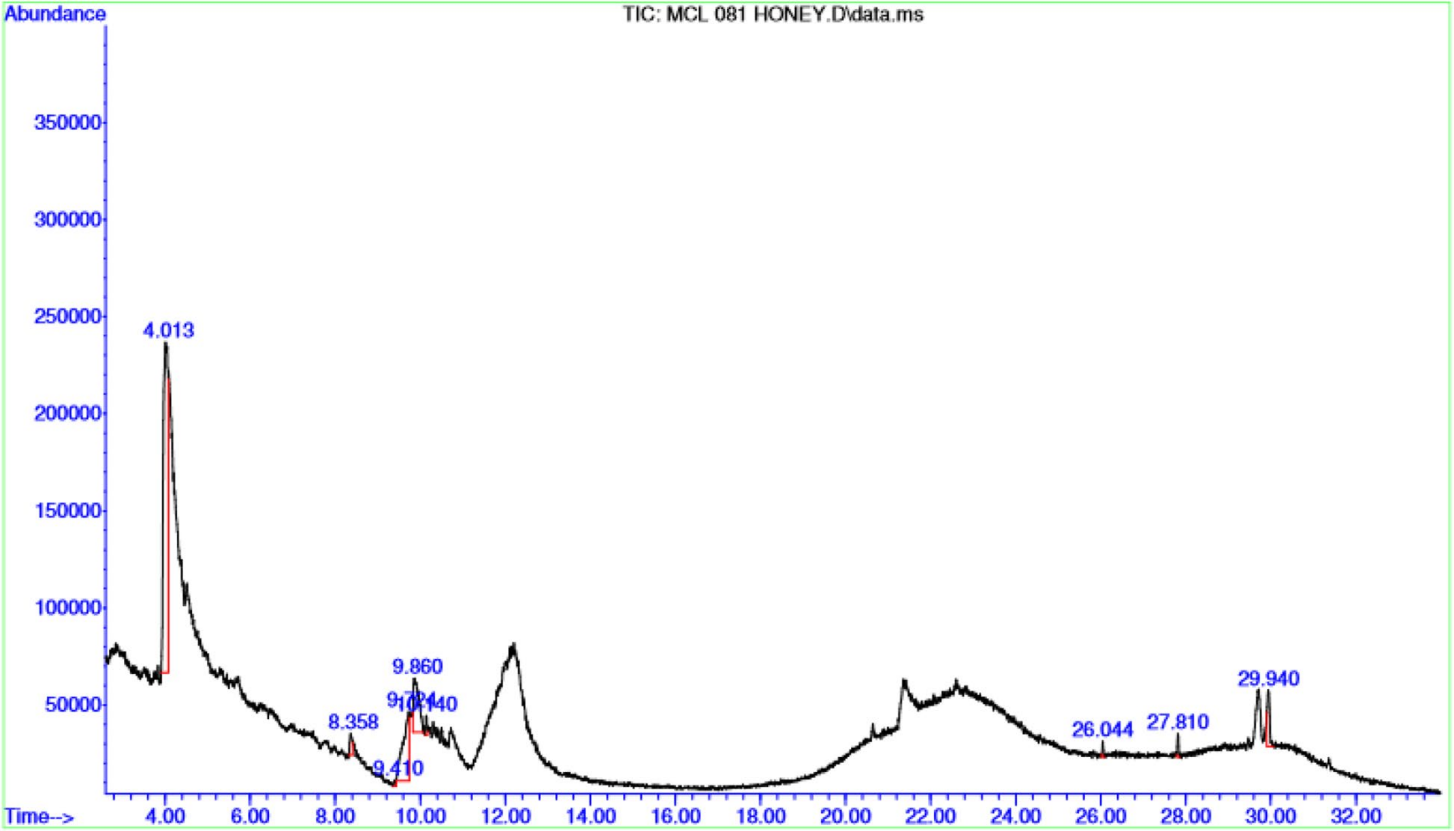
GC–MS spectrum of phytochemicals in honey

The 3-dimensional structure of the dihydropteroate synthase of *S. aureus* was obtained from the Research Collaboratory for Structural Bioinformatics (RCSC) protein database (Fig. 1). The properties of the retrieved NS2-3 and recorded (source, name of the protein, PDB ID, Uniprot name, Uniprot Accession ID, Uniprot taxonomic ID and organism were: sc-PDB, Dihydropteroate synthase, 1ad4, DHPS_STAAU, O05701, 1280, *Staphylococcus aureus*, respectively). Others were the protein resolution, and the default binding sites of the protein which were 2.400 and 33.1289, 7.9068 and 40.8571, respectively, for binding centres X, Y and Z [13].

### Assessment of ADMET and drug-likeness properties of the bioactive compounds

The drug-likeness and ADMET (absorption, distribution, metabolism, and excretion) properties of the bioactive compounds were evaluated as previously reported [24–29]. First, the canonical strings or the *S*implified Molecular-Input Line-Entry System (SMILES) of the various compounds were retrieved from PubChem. These strings were then used to evaluate the ADMET properties via the SWISSADME and the MCULE toxicity tools [13]. In addition to the ADMET parameters, other evaluated properties were metabolic half-life, bioavailability, oral absorption potential and permeability. Others were Lipinski’s rule of five, Egan, Muegge, Veber and the Ghose parameters (see supplementary results 1 and 2 for more details). In addition to these, the viability polygons were retrieved for all the ligands including that of the control were retrieved (see supplementary result 3).

### Docking analysis

The non-toxic bioactive compounds (those that met the Lipinski’s rule of five) were utilized in the docking analysis. The aforementioned default binding center was utilized for the binding. The retrieved 3-D structures of the ligands and protein were utilized for molecular docking. Molecular docking was done using the MCULE online tool. As a docking tool, it predicts the binding orientation or pose and the binding affinities (scores) of the various ligands against the dihydropteroate synthase. Following docking, the amino acid residues were recorded, and the binding poses retrieved for all the interactions and presented as previously reported [13]. The exclusion criterion for the selection of the binding poses was based on the highest docking score. The docking was further validated using the CB-dock tool (http://clab.labshare.cn/cb-dock/php/blinddock.php), a cavity guided blind docking tool and the docking pose retrieved as shown Figs. 3, 4, 5, 6, 7, 8, 9 and 10.

### Prediction of potential degradation product in human tissues and the gut microbiome

Prediction of potential degradation products was done using the Biotransformer software version 3.0 using the SMILES strings of the various ligands and the control (Djoumbou-Feunang et al., 2019 [30]. The option used for the prediction was the AllHuman that is capable of predicting the breakdown products in human tissues as well as gut microbiome. In addition, the mode chosen was the CYP450 combined mode that uses the rule-based method and the machine learned model and combines both results while the number of reactions iteraction selected was kept at 1.

### Data analysis

Resulting data from antimicrobial analysis were managed and analysed using Microsoft Excel version 2016. Data were converted into percentages.

## Results

**Fig. 2:**
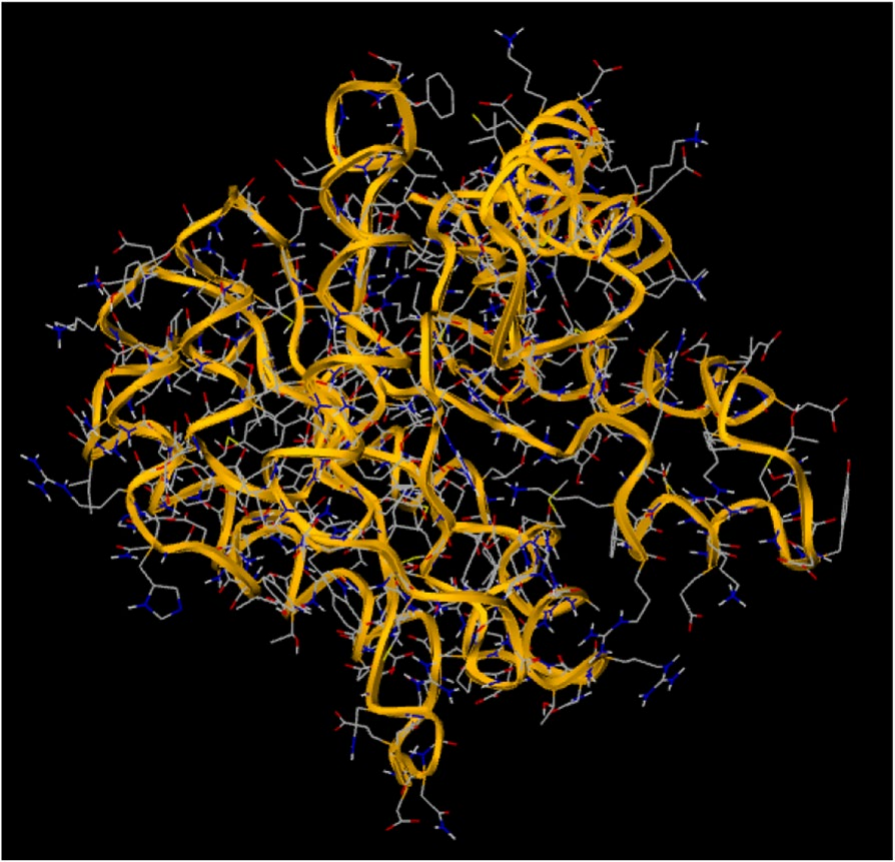
3-D of Dihydropteroate synthase of S. aureus

The phytochemicals in the honey sample used in our study is presented in Table 1. The GC_MS revealed a total of twenty (20) compounds that belonged to different categories of phytochemicals which included sugar (> 60%), organic acids, ether, to mention a few. The most abundant compound was to be Furfural. The second most abundant compound with a concentration of 11.75% was 2,3-Dihydro-3,5-dihydroxy-6-methyl-4 h-pyran-4-one. Figure 2 shows the GC–MS spectrum of the honey sample showing the peaks of the various compounds.

**Table 1:** Phytochemical screening analysis of honey

Phytochemical Group	Compounds	Concentration (%)	Toxicity
Sugar	Furfural	60.792	Yes
Cyclic ether Urea Cyclic ether Aldehyde Saturated fatty aldehyde	2,3- Epoxybutane	5.626	Yes
	N,N-Diethylurea	5.626	No
	2,3 – Epoxybutane	0.5125	Yes
	Octanal	5.847	Yes
	Heptanal	0.221	Yes
Purine	2R,3S-9-[1,3,4-Trihydroxy-2-butoxymethyl] guanine	1.742	Yes
Alcohol Modified pentose sugar	1,3- Cyclohexanediol	0.661	No
	D-erythro- Pentose, 2-deoxy	0.367	Yes
Alykne	6-Methyl-2-heptyne	0.661	No
	3-Buten-2-ol	0.192	Yes
Amine Amide Modified amino acid	N- Acetylethylenediamine	0.192	No
	Cyclopropanecarboxamide	0.221	No
	N-Acetyl-L-methionine	0.367	No
Ketone Heterocyclic compound	*4-Hydroxy-3-methyl-2-butanone*	0.192	Yes
	2,3-Dihydro-3,5-dihydroxy-6-methyl-4 h-pyran-4-one	11.753	Yes
	*2,3-Dihydro-3,5-dihydroxy-6-methyl-4 h-pyran-4-one*	0.5125	Yes
Organic acids	Succinic acid	1.742	No
	Fumaric acid	1.742	Yes
Organic acid esther	Carbamic acid, ethylnitroso- butyl ester	0.661	Yes
Secondary metabolite	Tetraacetyl-d-xylonic nitrile	0.367	Yes

The resulting twenty compounds from the GC–MS were first screened for potentially toxic functional groups using the MCULE toxicity checker. Following the screening, a total of 7 compounds as shown in Table 2 returned non-toxic side or functional groups. Table 2 further shows the Lipinski rule of five for the seven non-toxic compounds and they all obeyed the rule.

**Table 2:** Lipinski’s rule of five properties of bioactive compounds

S/N	CID	MW (g/mol) (≤ 500)	H Bond Acceptors (≤ 10.6)	H Bond Donors (≤ 5)	TPSA (A^2^) (< 40)	iLOGP (≤ 5)	Lipinski violations
1	N,N-Diethylurea	116.16	1	1	46.33	1.42	0
2	1,3- Cyclohexanediol	116.16	2	2	40.46	1.48	0
3	6-Methyl-2-heptyne	110.20	0	0	0.00	2.62	0
4	N- Acetylethylenediamine	102.14	2	2	22.15	0.95	0
5	Cyclopropanecarboxamide	85.10	1	1	43.09	0.89	0
6	N-Acetyl-L-methionine	191.25	3	2	91.70	1.03	0
7	Succinic acid	118.09	3	4	74.60	0.32	0
8	Thrimethoprim	380.42	6	2	130.47	2.07	0

The molecular weight (mw) of the selected compounds ranged from 85.10 to 191.25 g/mol. Compared to methicillin whose mw was 380.42 g/mol. The hydrogen bond acceptor for our bioactive compounds ranged from 0 to 3 while H bond donors ranged from 0 to 4. Furthermore, TPSA ranged from 0.00 to 91.70 while that of methicillin was 130.47, and was higher than those of the non-toxic bioactive compounds. The iLog P values ranged from 0.32 to 2.62 compared to that of methicillin which was 2.07. All the molecules were compliant with Lipinski’s rule of five.

Furthermore, the pharmacokinetics properties of the non-toxic compounds are presented in Table 3. All the bioactive compounds as well as trimethoprim all gave high gastrointestinal (GI) absorption status except for 6-Methyl-2-heptyne that gave low GI absorption. Also, the blood brain barrier permeabilities of the various compounds showed that they apart from 6-Methyl-2-heptyne showed ability to cross the blood brain barrier (BBB). All the substrates apart from trimethoprim were not potential substrate for P glycoprotein. All the bioactive compounds including the test antibiotic were not inhibitors of CYP1A2, CYP2CI9, and CYP2D6. The values for the logarithmic skin permeation coefficient ranged from -4.7 to -7.9 for the bioactive compounds and -7.7 for trimethoprim.

**Table 3:** Computed pharmacokinetic parameters of the screened compounds

S/N	CID	GI Absorption	BBB Permeability	PGP Substrate	CYP1A2 Inhibitor	CYP2CI9 Inhibitor	CYP2C9 Inhibitor	CYP2D6 Inhibitor	Log K_p_ (cm/s)
1	N,N-Diethylurea	High	No	No	No	No	No	No	-7.17
2	1,3- Cyclohexanediol	High	No	No	No	No	No	No	-6.86
3	6-Methyl-2-heptyne	Low	Yes	No	No	No	No	No	-4.71
4	N- Acetylethylenediamine	High	No	No	No	No	No	No	-7.92
5	Cyclopropanecarboxamide	High	No	No	No	No	No	No	-7.10
6	N-Acetyl-L-methionine	High	No	No	No	No	No	No	-7.43
7	Succinic acid	High	No	No	No	No	No	No	-7.44
8	Thrimethoprim	High	No	Yes	No	No	No	No	-7.75

Table 4 shows the results of the computed drug-likeness of the screened compounds and the test antibiotic. All the compounds met Lipinski’s rule of five, the Veber and Egan rules. However, two bioactive compounds violated the Muegge rule and only one bioactive compound violated the Ghose rules. Trimethoprim met all the drug-likeness rules. The bioavailability score for the bioactive compounds ranged from 0.55 to 0.85 while that of the antibiotic was 0.56.

**Table 4:** Computed Drug-likeness characteristics of the screened compounds

S/N	Bioactive compounds	Lipinski	Ghose	Veber	Egan	Muegge	Bioavailability Score
1	N,N-Diethylurea	0	No, 2	Yes	Yes	Yes	0.55
2	1,3- Cyclohexanediol	0	No, 2	Yes	Yes	No,1	0.55
3	6-Methyl-2-heptyne	0	No,2	Yes	Yes	No,2	0.55
4	N- Acetylethylenediamine	0	No,4	Yes	Yes	No, 2	0.55
5	Cyclopropanecarboxamide	0	No,3	Yes	Yes	No,2	0.55
6	N-Acetyl-L-methionine	0	Yes	Yes	Yes	No,1	0.56
7	Succinic acid	0	No,3	Yes	Yes	No,2	0.85
8	Thrimethoprim	0	Yes	Yes	Yes	Yes	0.56

### 3-D structure of dihydropteroate synthase

**Fig. 3:**
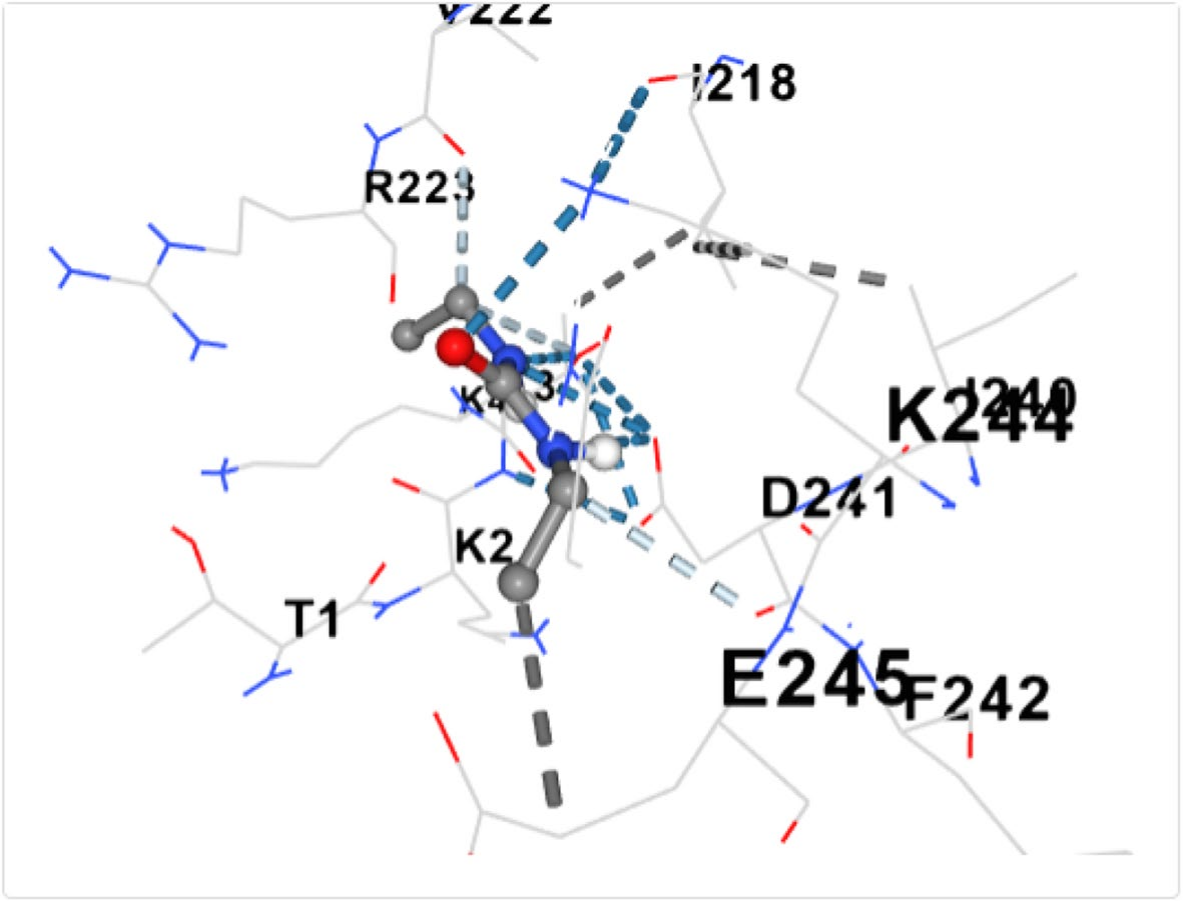
shows the docking of N,N-Diethylurea with dihydropteroate synthase of S. aureus

**Fig. 4:**
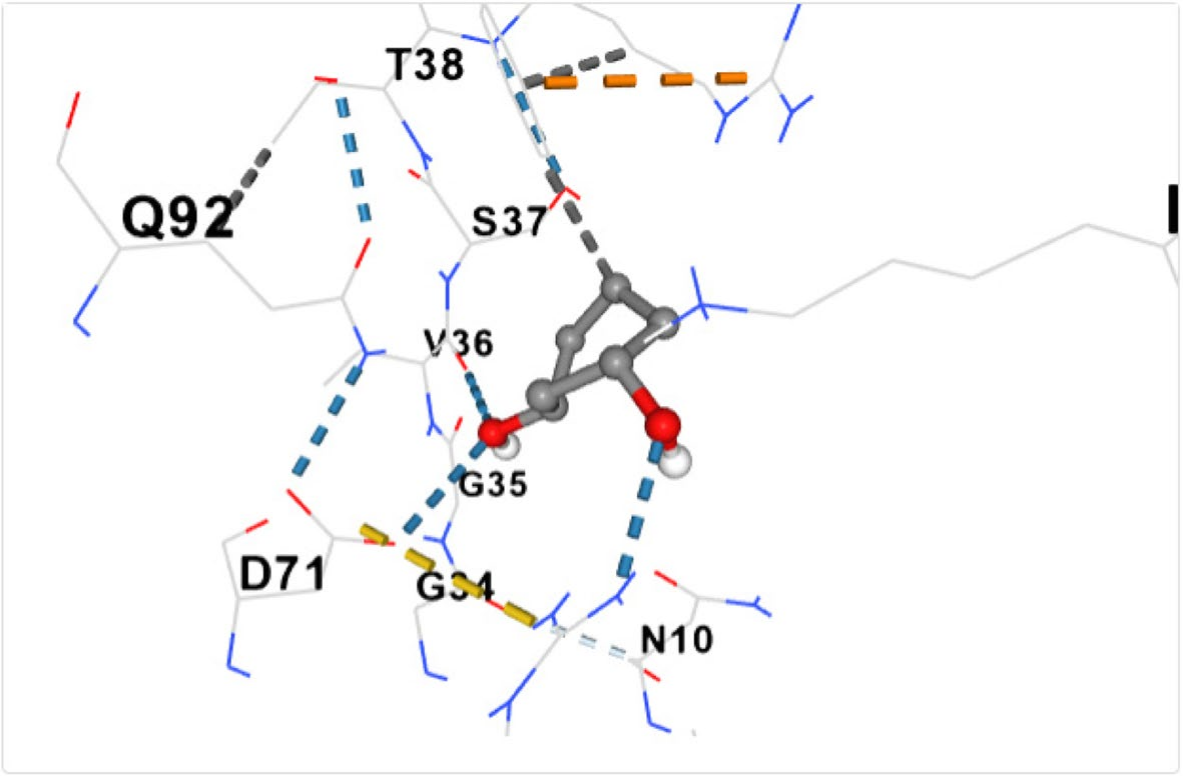
shows the docking of 1,3- Cyclohexanediol with dihydropteroate synthase of S. aureus

**Fig. 5:**
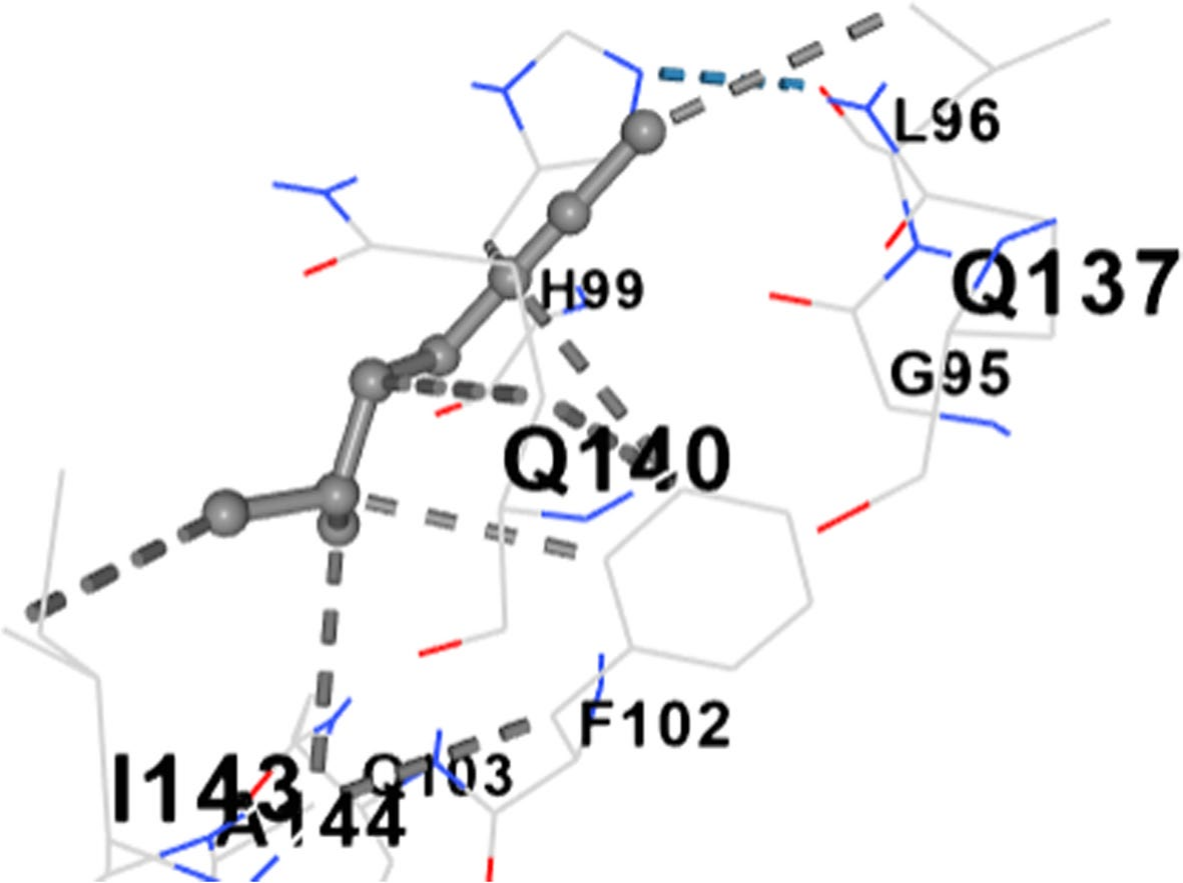
shows the docking of 6-Methyl-2-heptynewith dihydropteroate synthase of S. aureus

**Fig. 6:**
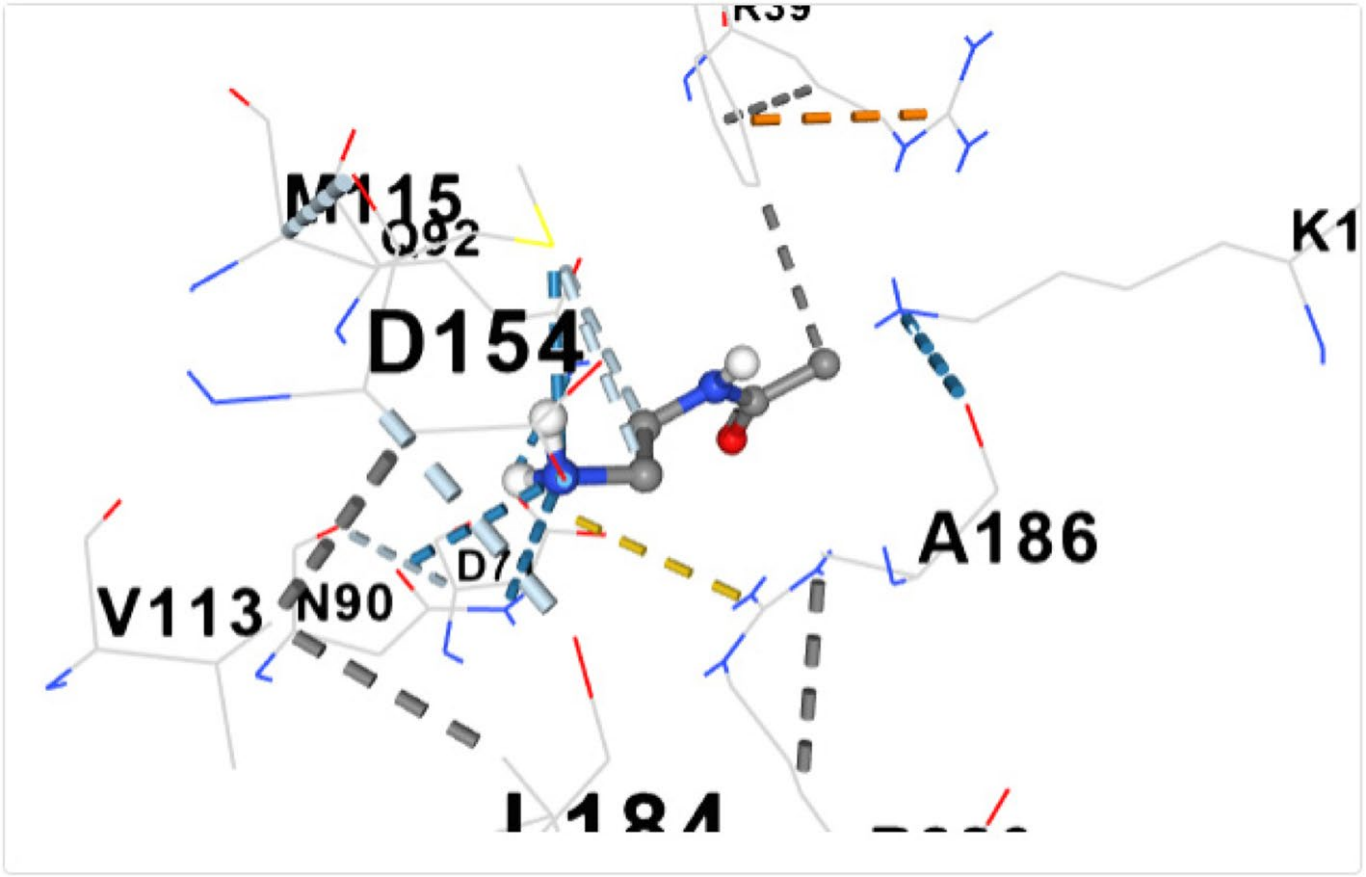
shows the docking of N- Acetylethylenediaminewith dihydropteroate synthase of S. aureus

**Fig. 7:**
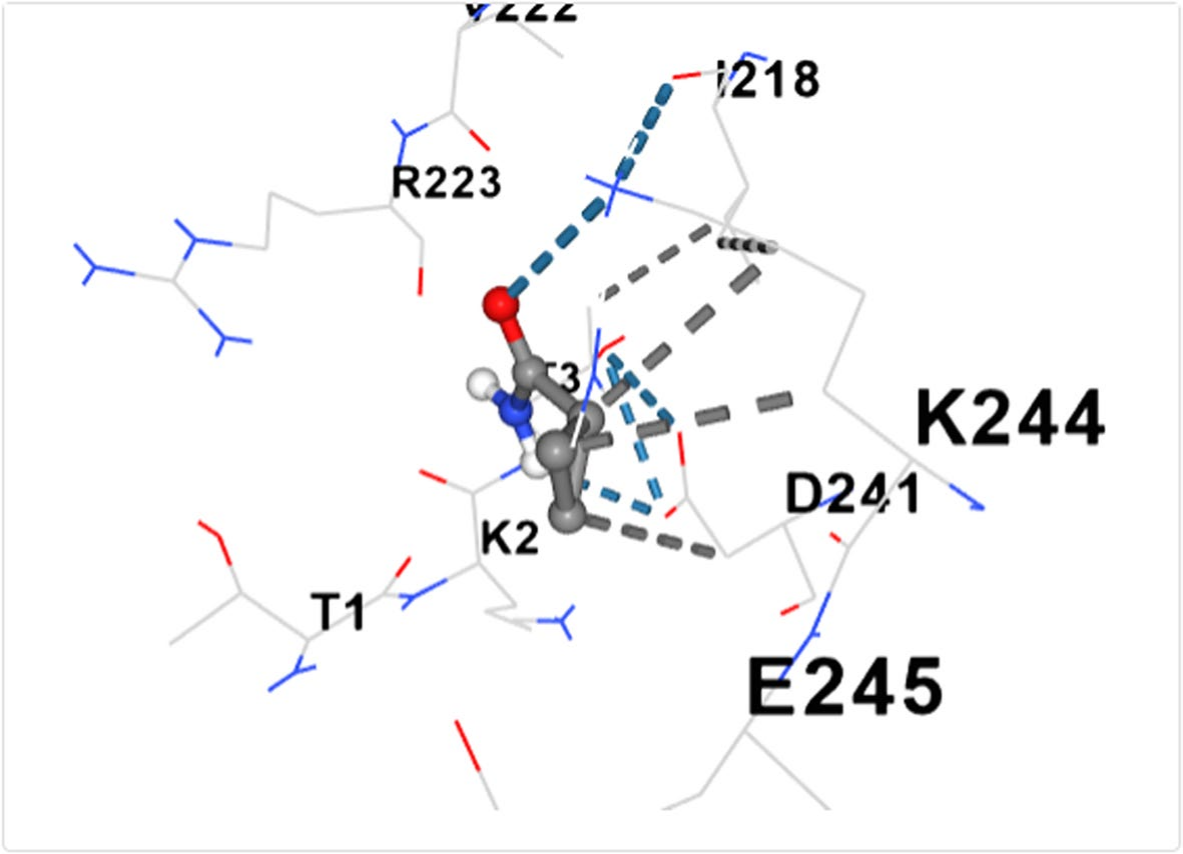
shows the docking of Cyclopropanecarboxamide with dihydropteroate synthase of S. aureus

**Fig. 8:**
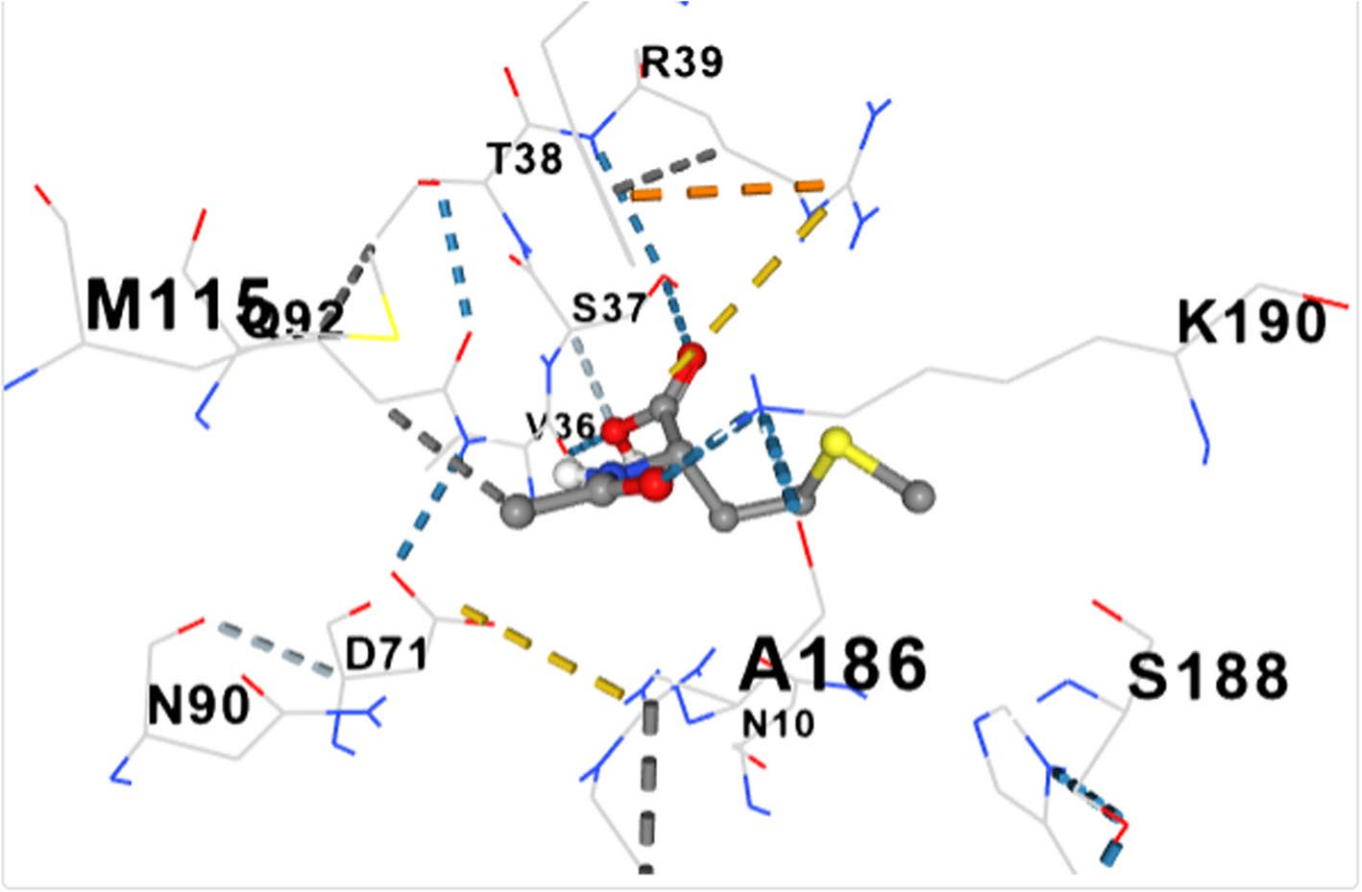
shows the docking of N-Acetyl-L-methionine with dihydropteroate synthase of S. aureus

**Fig. 9:**
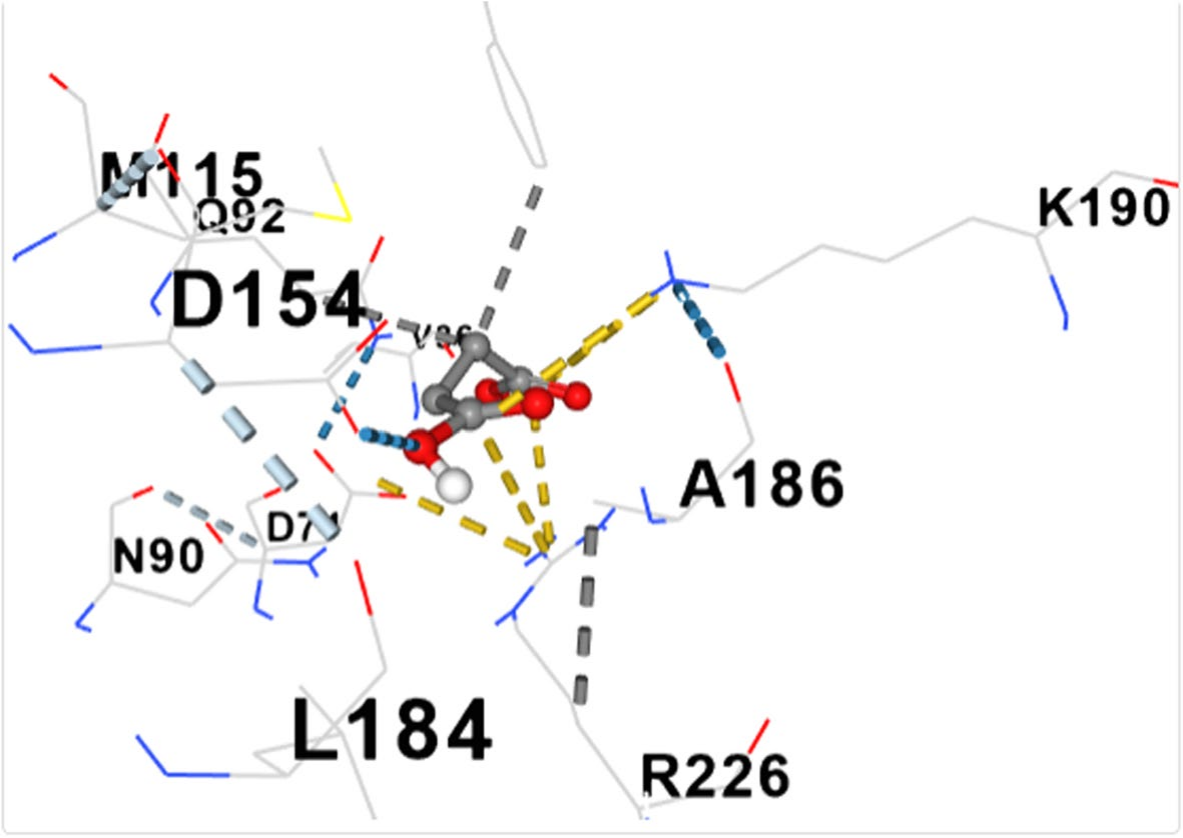
shows the docking of succinic acid with dihydropteroate synthase of S. aureus

**Fig. 10:**
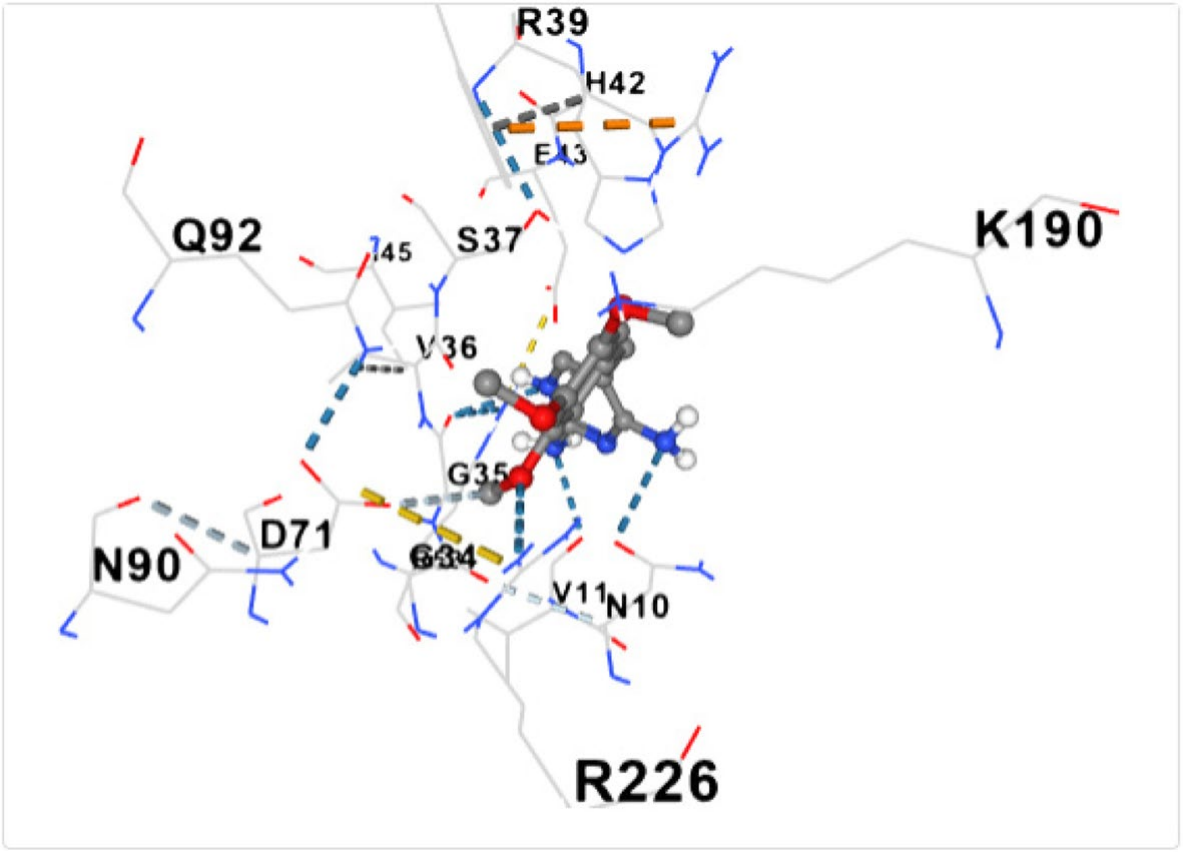
shows the docking of trimethoprim with dihydropteroate synthase of S. aureus

Figure 2 shows the 3-D structure of dihydropteroate synthase as retrieved from PDB. The active site of the protein was assessed using the active site predictor. The active site prediction revealed a total of 32 cavities. See supplementary data (Supplementary file 1). Figures 3, 4, 5, 6, 7, 8, 9 and 10 show the docking of N, N-Diethylurea1, 3- cyclohexanediol, 6-Methyl-2-heptyne, N- Acetylethylene diamine, cyclopropanecarboxamide, N-Acetyl-L-methionine, succinic acid and trimethoprim with dihydropteroate synthase of *S. aureus.*

Table 5 shows the amino acid residues and the best docking scores for the various bioactive compounds. The docking scores ranged from -3.6 to -4.5 for the bioactive compounds, and that of trimethoprim was -6.4, which was almost twice higher than N- Acetylethylenediamine which had a docking score of -3.6. The amino acid residues overlap among the bioactive compounds; however, some were unique to the trimethoprim. Both N-Acetyl-L-methionine and the control had 12 amino acid residues around their respective ligands. For both ligands, amino acid residues that were common were ASN90, ASN10, SER37, VAL36 and ASP71. These residues were held together by various that included weak hydrogen bond (light ash colour broken lines), hydrogen bond (deep blue broken line), cation pi bond (orange colour) and ionic interaction (deep broken as colour).

**Table 5:** Amino residues involved in binding of the various bioactive compounds against dihydropteroate synthase

Molecules	Amino acid residues	Docking/Vina scores (kcal/mole)
N,N-Diethylurea	K2 (LYS2), D243(ASP241), I218(ILE218), V222 (VAL222), R223 (ARG223), K4(LYS4), K244 (LYS244) and T3 (THE3)	-3.9
1,3- Cyclohexanediol	S37 (SER37), T38 (THR38), Q92 (GLN92), V36 (VAL36), G35 (GLY35), G34 (GLY34), D71 (ASP71) and N10 (ASN10)	-4.2
6-Methyl-2-heptyne	Q137 (GLN137), Q140 (GLN140), F102 (PHE102), I143 (ILE143), A144 (ALA144), G95 (GLY95), Q103 (GLN103), H99 (HIS99) and L96 (LEU96)	-3.9
N- Acetylethylenediamine	V113 (VAL133), N90 (ASN90), M115 (MET115), Q92 (GLY92), D154 (ASP154), A186 (ALA186), L184 (LEU184) and D7 (ASP77)	-3.6
Cyclopropanecarboxamide	K244 (LYS244), E245 (GLU244), T1 (THR1), K2 (LYS2), 1218 (ILE218), V222 (VAL222), T3 (THR3) and D241 (ASP241)	-3.7
N-Acetyl-L-methionine	M115 (MET115), Q92 (GLN92), N90 (ASN90), D71 (ASP 71), A186 (ALA186), S188 (SER188), K190 (LYS190), R39 (ARG223), T38 (THR38), S37 (SER37), V36 (VAL36) and N10 (ASN10)	-4.5
Succinic acid	M115 (MET115), S37(SER37), D154 (ASP154), N90 (ASN90), D7 (ASP7), A186 (ALA186), L184 (LEU184), R226 (ARG226), and K190 (LYS190)	-4.5
Trimethoprim (2,4-diamino-5-(3′,4′,5′-trimethoxybenzyl) pyrimidine)	N90 (ASN90), D71 (ASP71), N10 (ASN10), R226 (ARG226), V36 (VAL36), R39 (ARG39), S37 (SER37), K190(LYS190), G34 (GLY34), G35 (GLY35), H42 (HIS42) and I45 (ILE45)	-6.4

Table 6 shows the predicted breakdown down of the bioactive compounds, the reaction type and enzymes involved in the reactions. The various enzymes were cytochrome P450 1A2, cytochrome P450 2B6, Alcohol dehydrogenase, UDP-glucuronosyltransferase, Glycine N Acyltransferase, Phosphoglucomutase-1 and Uspecified microbial bile acid:amino acid N-acetyltransferase for the N,N-Diethylurea, 1,3- Cyclohexanediol, 6-Methyl-2-heptyne, cyclopropanecarboxamide, N-Acetyl-L-methionine, succinic acid and trimethoprim, respectively. The predicted breakdown products for the various bioactive compounds including the control were Ornithine, Ethyl urea and ethanal for N,N-Diethylurea, 3- Hydroxycyclohexanone for 1,3- Cyclohexanediol, cyclooctenone for 6-Methyl-2-heptyne, N-Glucuronidation of amide for cyclopropanecarboxamide, maleic acid for succinic acid, and 3'-Hydroxytrimethoprim; 5-[(2,4-Diaminopyrimidin-5-yl)methyl]-2,3-dimethoxyphenol for trimethoprim.

**Table 6:** Predicted degradation products of the bioactive compounds

Bioactive compounds	Product	Reaction type	Enzymes
N,N-Diethylurea	Ornithine	Hydroxylation of terminal methyl	Cytochrome P450 1A2
1,3- Cyclohexanediol	3- Hydroxycyclohexanone	Dehydrogenation of secondary alcohol	Alcohol dehydrogenase
6-Methyl-2-heptyne	Cyclooctenone	Hydroxylation of terminal methyl	Cytochrome P450 1A2
N- Acetylethylenediamine	NA	NA	NA
Cyclopropanecarboxamide	N-Glucuronidation of amide	N-Glucuronidation of amide	UDP-glucuronosyltransferase
N-Acetyl-L-methionine	Thiamet G	Glycine conjugation	Glycine N Acyltransferase
Succinic acid	Maleic acid	Dehydrogenation of (D)-2-hydroxy acid	Phosphoglucomutase-1
Trimethoprim (control)	3'-Hydroxytrimethoprim; 5-[(2,4-Diaminopyrimidin-5-yl) methyl]-2,3-dimethoxyphenol	Dephosphorylation of 5'-ribonucleotide	Unspecified microbial bile acid: amino acid N-acetyltransferase

Key: *NA* Not applicable

In addition to the various predicted metabolic products, we also predicted targets for the various bioactive compounds and the control and the results presented in Table 7. The various targets were family A G protein-coupled receptor, enzyme, cytochrome P450, oxidoreductase, other cytosolic protein and proteases, ligated-gated ion-channel, membrane receptor, cytochrome P450 and nuclear receptor among other. The highest targets were Family A G protein-coupled receptor (335) for N,N-Diethylurea, nuclear receptor (33%) for 1,3- cyclohexanediol, Enzymes (53.3) for 6-Methyl-2-heptyne, family A G protein-coupled receptor (26.7) for N-Acetylethylenediamine, eraser (33.3) for cyclopropanecarboxamide, protease (26.7) for N-Acetyl-L-methionine, ligand-gated ion channel (26.7) for succinic acid and kinase (40) for trimethoprim (control) (Supplementary file 4). The result of the viability polygon (See supplementary results 3), it can be seen that the control had the highest insolubility compared to all the bioactive compounds. Also, it recorded high instauration, polarity, flexibility, lipophilicity, and size. However, N-Acetyl-L-methionine was the closest to the control in terms of the properties in the viability polygon.

**Table 7:** Predicted targets for the various bioactive compounds and control

Bioactive compounds	Targets (%)
N,N-Diethylurea	Family A G protein-coupled receptor (33), enzyme (20), Cytochrome P450 (6.70), oxidoreductase (20), other cytosolic protein (6.7) and proteases (15.30)
1,3- Cyclohexanediol	Other membrane proteins (6.7), voltage gated ion channel (6.7), enzymes (6.7), nuclear receptor (33), lyase (13.3), phosphatase (13.3), secreted protein (6.7), protease (6.7) and family A G protein-coupled receptor (6.7)
6-Methyl-2-heptyne	Enzyme (53.3), family A G protein-coupled receptor (20), ligated-gated ion-channel (6.7), membrane receptor (6.7), cytochrome P450 (6.7) and nuclear receptor (6.7)
N- Acetylethylenediamine	Ligand-gated ion channel (20), writer (20), enzymes (6.7), cytochrome P450, protease (20) and family A G protein-coupled receptor (26.7)
Cyclopropanecarboxamide	Oxidoreductase (13.3), eraser (33.3), enzyme (13.3), protease (6.7), kinase (6.7), phosphatase (6.7), other cytoplasmic protein (6.7) and hydrolase (13.3)
N-Acetyl-L-methionine	Family A G protein-coupled receptor (6.7), kinase (6.7), phosphatase (6.7), protease (26.7), lyase (13.3), membrane receptor (6.7) and enzyme (20)
Succinic acid	Oxidoredutase (6.7), electrochemical transporter (26.7), eraser (13.3), ligand-gated ion channel (26.7), enzymes (20) and family A G protein-coupled receptor (6.7)
Trimethoprim (control)	Oxidoreductase (6.7), enzymes (20), other cytosolic protein (6.7), kinase (40), phosphodiesterase (6.7), family A G protein-coupled receptor (6.7) and protease (6.7)

Table 8 shows the comparative susceptibility of the honey and trimethoprim to the various *S. aureus* isolates to different concentrations of honey and trimethoprim while Table 9 shows the summary of the various sensitivities of the isolates to various concentrations of honey and trimethoprim. The concentrations used were neat (100%), 1:2 (50%), 1:4 (25%), 1:8 (12.5%), 1:16 (6.25%), 1:32 (3.13%), 1:64 (1.57%), 1:128 (0.79%) and 1:258 (0.40%). At neat concentration, all the isolates were 100% sensitive except for 1 from patient number 6. Other concentrations, that is, 1:2; 1:4, 1:8, 1:16, 1:32, 1:64, 1:128, and 1:256 showed an increasing level of resistance that was almost 100% for all the isolates except for three isolates from patient 1 that were sensitive. For trimethoprim, the various concentrations used were 0.39, 0.78, 1.56, 3.13, 6.25, 12.50, 25.0, 50.0 and 100 mg/ml. At concentrations of 1:32 to 1:256, at least 65.96% of the isolates were resistant to honey while for trimethoprim at 3.13 l% to 0.39 mg/ml, the percentage of resistance went from 57.45 to 100%.

**Table 8:** Comparative susceptibility profile of *S. aureus* species to different concentrations of honey

Susceptibility to different concentrations of honey																		
	**Neat (100%)**		**1:2 (50%)**		**1:4 (25%)**		**1:8 (12.5%)**		**1:16 (6.25%)**		**1:32 (3.13%)**		**1:64 (1.57%)**		**1:128 (0.79%)**		**1:256 (0.40%)**	
**Test organisms (*n* = 47)**	**S**	**R**	**S**	**R**	**S**	**R**	**S**	**R**	**S**	**R**	**S**	**R**	**S**	**R**	**S**	**R**	**S**	**R**
**Patient 1 (20)**	20 (100.0)	0 (0.0)	18 (90.0)	2 (10.0)	17 (85.0)	3 (15.0)	16 (80.0)	4 (20.0)	15 (75.0)	5 (25.0)	13 (65.0)	7 (35.0)	9 (45.0)	11 (55.0)	6 (30.0)	14 (70.0)	3 (15.0)	17 (85.0)
**Patient 2 (6)**	6 (100.0)	0 (0.0)	6 (100.0)	0 (0.0)	4 (67.0)	2 (33.0)	4 (67.0)	2 (33.0)	3 (50.0)	3 (50.0)	2 (33.0)	4 (67.0)	1 (16.7)	5 (83.3)	0 (0.0)	6 (100.0)	0 (0.0)	6 (100.0)
**Patient 3(7)**	7 (100.0)	0 (0.0)	6 (85.7)	1 (14.3)	6 (85.7)	1 (14.3)	5 (71.4)	2 (28.6)	4 (57.1)	3 (42.9)	3 (42.9)	4 (57.1)	2 (28.6)	5 (71.4)	1 (14.3)	6 (85.7 l)	0 (0.0)	7 (100.0)
**Patient 4(5)**	5 (100.0)	0 (0.0)	4 (80.0)	1 (0.0)	4 (80.0)	1 (20.0)	3 (60.0)	2 (40.0)	2 (40.0)	3 (60.0)	1 (20.0)	4 (80.0)	0 (0.0)	5 (100.0)	0 (0.0)	5 (100.0)	0 (0.0)	5 (100.0)
**Patient 5(2)**	2 (100.0)	0 (0.0)	2 (100.0)	0 (0.0)	2 (100.0)	0 (0.0)	2 (100.0)	0 (0.0)	1 (50.0)	1 (50.0)	1 (50.0)	1 (50.0)	0 (0.0)	2 (100.0)	0 (0.0)	2 (100.0)	0 (0.0)	2 (100.0)
**Patient 6(7)**	6 (100.0)	0 (0.0)	6 (85.7)	1 (14.3)	5 (71.4)	2 (28.6)	3 (42.9)	4 (57.1)	2 (28.6)	5 (71.4)	2 (28.6)	5 (71.4)	1 (14.3)	6 (85.7)	0 (0.0)	7 (100.0)	0 (0.0)	7 (100.0)
**Susceptibility to different concentrations of trimethoprim**																		
	**0.39 mg/ml**		**078 mg/ml**		**1.56 mg/ml**		**3.13 mg/ml**		**6.25 mg/ml**		**12.50 mg/ml**		**25.0 mg/ml**		**50.0 mg/ml**		**100 mg/ml**	
Test organisms (*n* = 47)	**S**	**R**	**S**	**R**	**S**	**R**	**S**	**R**	**S**	**R**	**S**	**R**	**S**	**R**	**S**	**R**	**S**	**R**
**Patient 1 (20)**	0 (0.0)	20 (100.0)	2 (10.0)	18 (90.0)	5 (25.0)	15 (75.0)	9 (45.0)	11 (55.0)	14 (70.0)	6 (30.0)	17 (85.0)	3 (15.0)	18 (90.0)	2 (10.0)	20 (100.0)	0 (0.0)	20 (100.0)	0 (0.0)
**Patient 2 (6)**	0 (0.0)	6 (100.0)	0 (0.0)	6 (100.0)	1 (16.7)	5 (83.3)	2 (33.3)	4 (66.7)	4 (66.67)	2 (33.3)	6 (100.0)	0 (0.0)	6 (100.0)	0 (0.0)	6 (100.0)	0 (0.0)	6 (100.0)	0 (0.0)
**Patient 3 (7)**	0 (0.0)	7 (100.0)	0 (0.0)	7 (100.0)	2 (28.6)	5 (71.4)	3 (42.9)	4 (57.1)	3 (42.9)	4 (57.1)	7 (100.0)	0 (0.0)	7 (100.0)	0 (0.0)	7 (100.0)	0 (0.0)	7 (100.0)	0 (0.0)
**Patient 4 (5)**	0 (0.0)	5 (100.0)	0 (0.0)	5 (100.0)	1 (20.0)	4 (80.0)	2 (40.0)	3 (60.0)	4 (80.0)	1 (20.0)	5 (100.0)	0 (0.0)	5 (100.0)	0 (0.0)	5 (100.0)	0 (0.0)	5 (100.0)	0 (0.0)
**Patient 5 (2)**	0 (0.0)	2 (50.0)	0 (0.0)	2 (50.0)	0 (0.0)	2 (100.0)	1 (50.0)	1 (50.0)	0 (0.0)	2 (50.0)	2 (100.0)	0 (0.0)	2 (100.0)	0 (0.0)	2 (100.0)	0 (0.0)	2 (100.0)	0 (0.0)
**Patient 6 (7)**	0 (0.0)	7 (100.0)	0 (0.0)	7 (100.0)	2 (28.6)	5 (71.4)	3 (42.9)	4 (57.1)	4 (57.1)	3 (42.9)	7 (100.0)	0 (0.0)	7 (100.0)	0 (0.0)	7 (100.0)	0 (0.0)	7 (100.0)	0 (0.0)

**Table 9:** Summary of the sensitivities of the isolates to various concentrations of honey and trimethoprim

Agent/ dilution	Number of sensitive species	Number of resistant species	% Sensitive	% Resistant
**Honey**				
Neat	47	0	100.0	0.00
1:2	42	5	89.36	10.64
1:4	38	9	80.85	19.15
1:8	33	14	70.20	29.80
1:16	27	20	57.50	42.50
1:32	16	31	34.04	65.96
1:64	14	33	29.78	70.21
1:128	7	40	14.89	85.11
1:256	3	44	6.38	93.61
**Thrimethoprim (mg/ml)**				
100	47	0	0.00	100.0
50	47	0	0.00	100.0
25	45	2	95.74	4.26
12.5	44	3	93.61	6.38
6.25	29	18	61.70	38.30
3.13	20	27	42.55	57.45
1.56	2	45	4.26	95.74
0.78	2	45	4.20	95.80
0.39	0	47	0.00	100.00

## Discussion

We evaluated the antimicrobial property of honey and that of trimethoprim on *S. aureus* isolates from wound samples of patients. In addition, we examined the bioactive compounds of the honey samples and performed molecular docking of the bioactive compounds against the dihydropteroate synthase of *Staphylococcus aureus.* The honey sample used in our study returned via GC–MS twenty (20) compounds that belonged to various categories of compounds that included alcohol, modified sugar/amino acid, sugars, organic acids, amine, amides, and alkynes among other components. The most abundant compounds were various forms of sugar and this was followed by cycle ether. The composition of our study honey is in line with previous reports that showed honey is a multi-component substance [16} even though its composition is known to vary with seasons and locations [15–17]. The various components and physical properties of honey make it an excellent antimicrobial substance and this explains its use since time immemorial (in folk medicine) in the management of chronic wounds infected by microorganisms [31–33]. *S. aureus* is among the top four colonizers of wounds [10, 14]. As a potential pathogen, it is a very successful one due to its abilities to outwit the human immune system, form biofilms and acquire resistance genes with ease [10, 13, 14].

In this study, the isolates showed varying levels of antimicrobial sensitivity and resistance that were concentration dependent. The isolates were very sensitive to the top three concentrations for both honey (Neat,1:2 and 1:4) and trimethoprim (100 mg/ml, 50 mg/ml and 25 mg/ml) used in this study. As the concentrations of the honey and trimethoprim increased, the resistance of the isolates decreased such that at the least three concentrations of honey, the resistance levels ranged from 70.21 to 93.61% and 95.74 to 100.00%, respectively. Isolation of MDR isolates from wounds has been reported in numerous studies [34–37]. Älgå et al*.* [32] observed MAR in 36 out of 49 (73%) patients whose wounds were infected in their study in Syria. Khandia et al*.* [34] presented the first-ever report of MDR *C. perfringens* single isolate that showed resistance to harbouring resistance against at least 40 antibiotics tested in India. Furthermore, Gedebo et al. [33] observed that 86.2% of their isolated *S.aureus* and 28.6% of Coagulase negative *Staphylococci* became MDR in Ethiopia. The prevalence of antibiotic resistance in infections is known to have both adverse clinical and economic outcomes [34, 37, 38]. These adverse outcomes from MDR have promoted the search for alternatives such as plants and honey [3, 15, 32].

Honey was the mainstay of wound healing before the arrival of antibiotics [33, 34]. In addition to the evaluation of its antimicrobial properties, we evaluated the ADMET properties of the 20 bioactive compounds identified by GC–MS and only seven meet Lipinski’s rule of five and these were N, N-Diethylurea, 1,3- Cyclohexanediol, 6-Methyl-2-heptyne, N- Acetylethylenediamine, cyclopropane carboxamide, N-Acetyl-L-methionine and succinic acid. We evaluated their lead compounds against dihydropteroate synthase of *Staphylococcus aureus* with trimethoprim as a positive control using the default binding sites: 33.12, 7.91 and 40.86 for X, Y and Z axes respectively. The docking results did show that the bioactive compounds showed better affinity to the dihydropteroate synthase as it returned better dockings scores that were in the range of -3.3 to -4.6 compared to that of trimethoprim which was -6.1, indicating better binding or interacting with the dihydropteroate synthase [39].

Eukaryotes depend on dietary intake of folic acid, unlike prokaryotes that can synthesize their folic acid using dihydropteroate synthase [40]**,** an important enzyme that is the target of the sulfa drugs is a key enzyme that catalyzes the conversion of para-aminobenzoate to dihydropteroate [41]. The bioactive compounds interacted with dihydropteroate synthase revealing common and unique amino acid residues. The amino acid residues VAL36 and MTH115 were common for at least four bioactive compounds. Our common amino acid residues differed from an earlier study which examined dihydropteroate synthase of *Escherichia coli* and *S. aureus* against various compounds using almost similar docking coordinates [41]. All the bioactive compounds in our study met the Lipinski rule of five, the Veber and Egan rule further confirming their lead compound potential [27, 42, 43]. Compared to trimethoprim, all the bioactive compounds were not substrate to p-glycoproteins and thus, further enhancing their gastrointestinal absorption [42–44]. Despite the broad substrate specificity of CYP1A2, a compound known to aid the excretion of 5% of commercially available drugs, none of the bioactive compounds were potential inhibitors of CYP1A2, further enhancing their bioavailabilities and adverting the potential for herb-drug interaction [45, 46]. Similarly, they were also not also inhibitors of the P450 cytochrome enzymes CYP2CI9 and CYP2D6 [47, 48].

The human gut microbiome and tissues produce a number of non-essential metabolites. These metabolites are generated via the process of activation, detoxification and elimination of metabolic by-products or xenobiotics [30, 49]. Humans are exposed to a huge number of chemicals for which pharmaceuticals are also included and these are largely uncharacterized [49]. Metabolism of drugs or xenobiotics is known to significantly influence their pharmacokinetics and pharmacodynamics and their derivatives within a biological system [50]. The predicted breakdown products from the bioactive compounds and the control showed differences in terms of the reaction pathways and products. The predicted breakdown products were catalyzed by four different categories of enzymes and these were the P450 1A2 cytochrome enzyme (2 bioactive compounds), the transferases (three bioactive compounds), and the mutase and dehydrogenase families (1 bioactive compound) each. One of the bioactive compounds (N- acetyl ethylenediamine) did not return any result. Those catalyzed by the cytochrome P450 1A2 enzyme are those that are predicted to be excreted via phase I that both involved hydroxylation of terminal methyl in N,N-Diethylurea and 6-Methyl-2-heptyne into ornithine and cyclooctenone. The cytochrome enzymes can be induced or inhibited and therefore caution is needed when administering drugs that are metabolizable by the cytochrome enzymes [51, 52], and interestingly, our bioactive compounds as revealed by the ADME properties are not inhibitors of CYP1A2, CYP2CI9 and CYP2D6. For the other compounds, including the control, the predicted breakdown product appears to be obtained via phase II that involves the removal or transfer of various polar groups such as amino acids as shown by the N-Glucuronidation of amide, dephosphorylation of 5'-ribonucleotide and glycine conjugation predicted reactions [52]. These derivatives are capable of altering the efficacy of a drug positively or negatively and as seen with the bioactive compounds utilizing the phase I pathways, their administration need to be performed with caution [52].

## Conclusion

The science of wound care is evolving, and the search for an ideal compound to combat the incidence and menace of multi-drug-resistant pathogens is increasing. The *in-vitro* antimicrobial analysis of honey revealed similar levels of resistance and sensitivity at different concentrations. GC–MS revealed a total of twenty compounds that were majorly sugars. ADMET analysis revealed seven compounds with favourable pharmacokinetic properties comparable to trimethoprim. The bioactive compounds returned binding scores that were comparable to trimethoprim. Furthermore, the bioactive compounds were not inhibitors of the various cytochrome P459 proteins (CYP1A2, CYP2CI9 and CYP2D6) and p-glycoproteins, further enhancing their bioavailability. The various bioactive compounds showed various breakdown products via phase I and II pathways. Our findings suggest that one of the possible mechanisms of action of the bioactive compounds of honey could be via the blocking of dihydropteroate synthase in *S. aureus*.

## Supplementary Information

**Additional file 1:**

## Data Availability

All data generated or analysed during this study are included in this published article [and its supplementary information files.

## Abbreviations

ADMET: Absorption, distribution, metabolism, excretion and toxicity
BBB: Blood brain barrier
GC-MS: Gas chromatography-mass spectrophotometer
MAR: Multi-drug resistance
MSA: Mannitol salt agar
MW: Molecular weight
NIST: National Institute Standard and technique
RCSC: Research Collaboratory for Structural Bioinformatics
SMILES: *S*Implified Molecular-Input Line-Entry System
WHO: World Health Organization
^o^C: Degree Celsius
mins: Minutes
hrs: Hours
mm: Millimeter
%: Percentage
mg/ml: Milligram/milliliters
3-D: 3-Dimensional
