# Supplementary figures and images for: Antimicrobial analysis of honey against Staphylococcus aureus isolates from wound, ADMET properties of its bioactive compounds and in-silico evaluation against dihydropteroate synthase
Source: BMC Complement Med Ther. 2023 Feb 6;23:39. doi: 10.1186/s12906-023-03841-z (PMC9901111; doi:10.1186/s12906-023-03841-z)

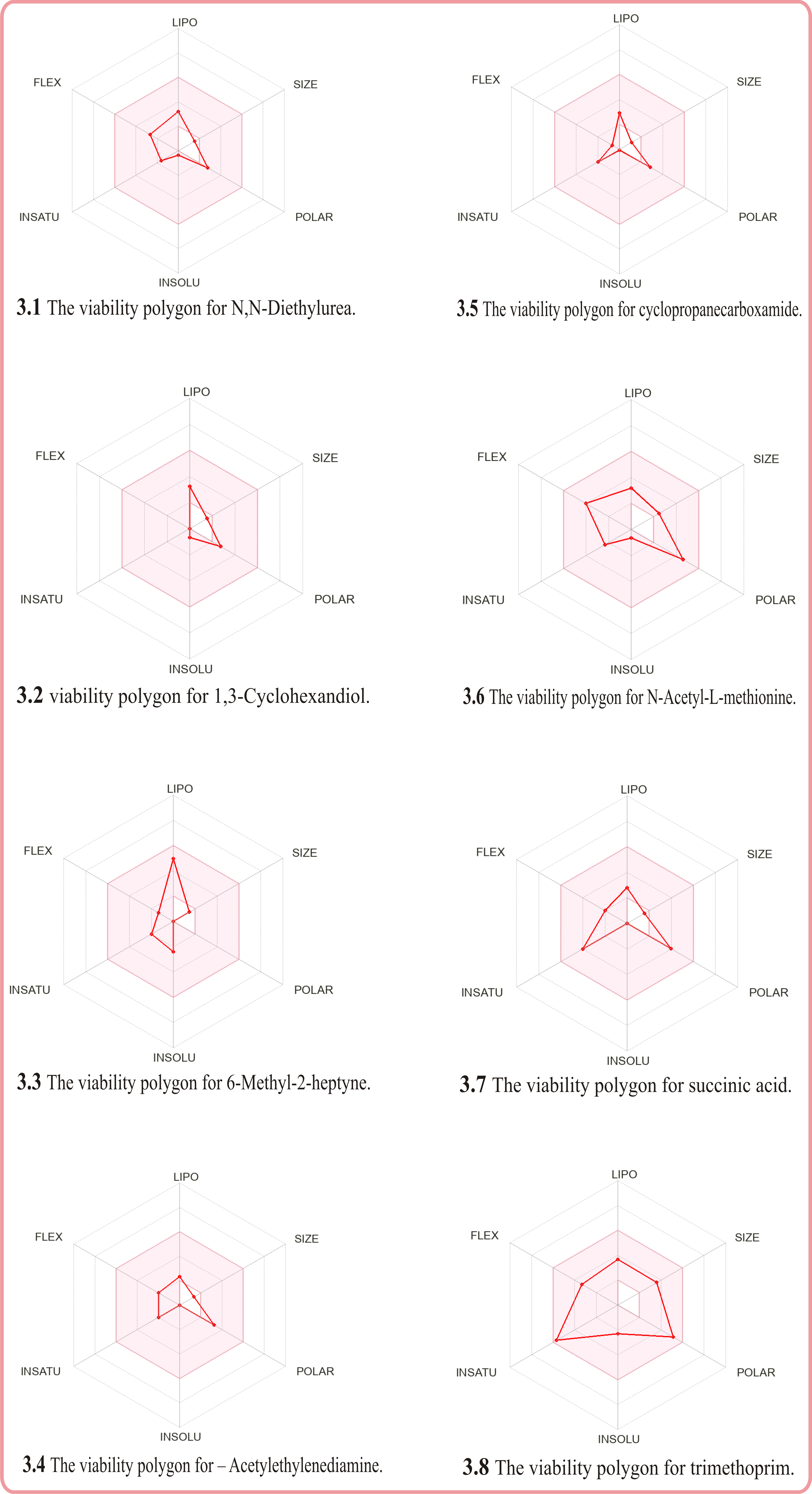

Supplement: Supplementary file 1 — Additional file 1: [file 12906_2023_3841_MOESM1_ESM.zip › supplementary result 3.png]

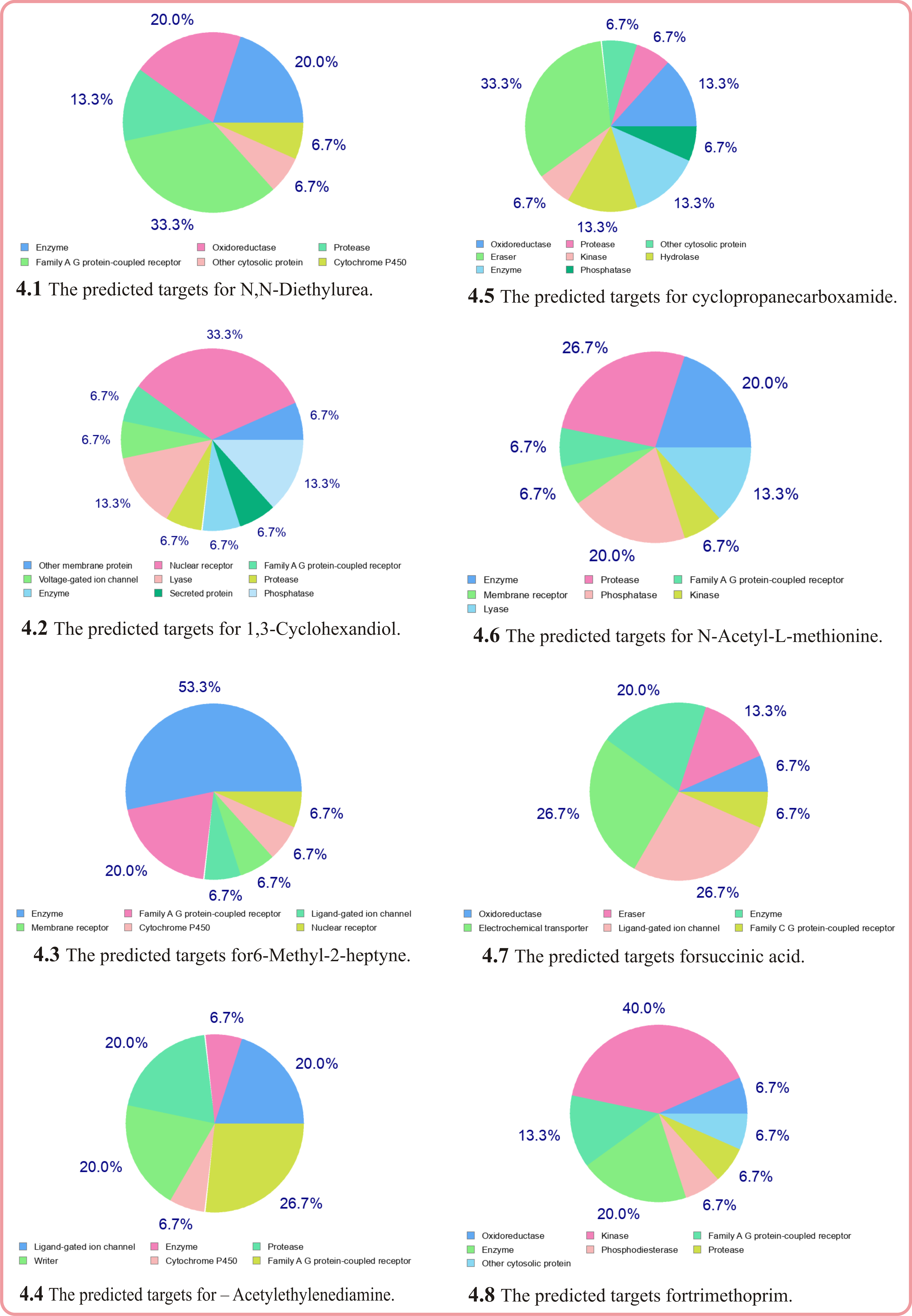

Supplement: Supplementary file 1 — Additional file 1: [file 12906_2023_3841_MOESM1_ESM.zip › supplementary result 4.png]
